# Association between the HOTAIR polymorphisms and cancer risk: an updated meta-analysis

**DOI:** 10.18632/oncotarget.13880

**Published:** 2016-12-10

**Authors:** Zhao-Xiong Zhang, Xue Tong, Wan-Ni Zhang, Wei-Neng Fu

**Affiliations:** ^1^ Department of Medical Genetics, China Medical University, Shenyang, 110122, P.R. China

**Keywords:** HOTAIR, SNP, cancer, susceptible risk, meta-analysis

## Abstract

**Purpose:**

LncRNA HOTAIR plays an important role in many cancer. Several studies have shown that some HOTAIR SNPs might be associated with tumor risk in case-control studies, but the results are inconsistent and inconclusive. Therefore, it is necessary to better evaluate association between the HOTAIR SNPs and the risk of cancer.

**Results:**

rs920778, rs7958904 and rs874945 but not rs4759314 and rs1899663 loci were significantly related to cancer risk, among of which rs920778 and rs874945 increased and rs7958904 decreased cancer risk, respectively. Moreover, rs920778 is significantly susceptible in both Asian population and digestive cancer risks.

**Materials and Methods:**

Data were collected from PubMed, Embase and Web of Science. A total of 11 case-control studies were selected for the quantitative analysis. Software Stata (Version 12) was used to calculate Odds ratios (ORs) and 95% confidence intervals (CIs) to evaluate the strength of the associations. Subgroup analysis, sensitivity analysis, and publication bias were also performed. Five HOTAIR SNPs were finally enrolled in the study.

**Conclusions:**

HOTAIR SNP rs920778, rs7958904 and rs874945 are susceptible to cancer risk. SNP rs920778 is also a useful risk factor in evaluation of Asian population and digestive cancer. In addition, the cancer risk SNP rs874945 is first reported in the meta-analysis.

## INTRODUCTION

Long non coding RNAs (LncRNAs) are a class of regulatory RNAs that are longer than 200 nucleotides and lack protein coding capacity [1]. LncRNAs play critical roles in physiologic and pathologic processes, including carcinogenesis [2, 3]. In addition, lncRNAs are also involved in cellular processes such as differentiation [4], proliferation [5], apoptosis [6], metabolism [7] and autophagy [8].

Recently, genome-wide association studies (GWAS) have revealed a large number of genetic variants related to different types of cancer. For examples, Guo et al. identified 45 candidate lncRNAs regulated by noncoding SNPs in prostate cancer [9]. Yuan et al. found that a novel SNP rs114020893 in the lncRNA NEXN-AS1 gene is significantly associated with an increased risk of lung cancer [10]. However, there are some limitations in the GWAS. For examples, at least one-third of the identified variants in non-coding intervals regulatory regions modulate transcription factor binding [11, 12], but the relationship between phenotype-related loci and lncRNAs is largely unknown. Furthermore, many GWAS results have shown substantial heterogeneity in allele frequencies across different population [13]. Therefore, further study on a single candidate lncRNA gene in the development of cancer is necessary.

LncRNA HOTAIR, which is located within the Homeobox C (HOXC) gene cluster on chromosome 12 and is co-expressed with HOXC genes, is a inhibitor in the HOXD gene transcription [14]. As an oncogene, HOTAIR is overexpressed in many cancer and involved in cancer proliferation, migration, invasion, progression and poor prognosis, suggesting that it might be a potential novel target in cancer therapy [15, 16].

As a class of genetic variants, SNPs are widely used in prediction of disease risk [17], prognosis [18] and clinical outcome [19]. Recently, several studies have summarized the associations of HOTAIR SNPs with cancer risk. However, some results are controversial. For example, two groups reported that there is no association between rs4759314 polymorphism and cancer risk [20, 21], but Qi et al. found that rs4759314 is in association with cancer risk [22]. Obviously, the association analysis between HOTAIR SNPs and cancer susceptibility is still necessary.

In the study, a total of 5 SNPs collected from 11 articles were finally enrolled for meta-analysis, three of which were significantly associated with cancer risk, suggesting they are important cancer risk factors and potential targets in future clinical study.

## RESULTS

### Characteristics of eligible studies

**Figure 1:**
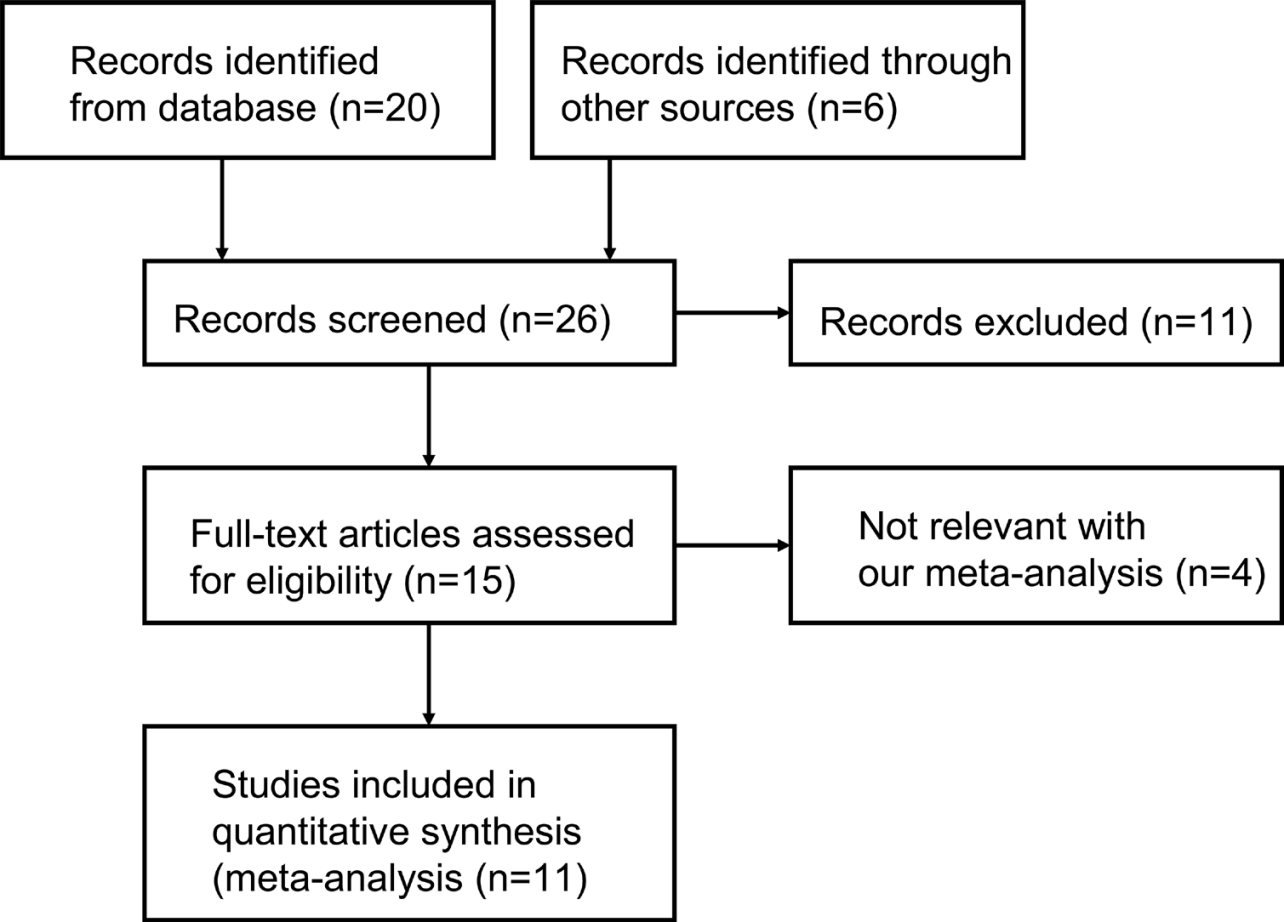
Flow diagram summarizing the selection of eligible studies

A total of 26 articles on relationship between HOTAIR SNPs and cancer risk were retrieved after first search in PubMed, Embase and Web of Science. As shown in Figure 1, 11 case-control publications including 10867 patients and 13172 controls met the inclusion criteria and 5 HOTAIR SNPs were involved in the meta-analysis [23–33]. The main characteristics of 5 HOTAIR SNPs were listed in Table 1. Of the 11 studies, the HOTAIR rs4759314 in 8 studies [23–25, 27, 29–32], rs920778 in 6 studies [23, 26–28, 30, 33], rs7958904 in 4 studies [24, 29, 31, 32], rs874945 in 4 studies [24, 29, 31, 32] and rs1899663 in 3 studies [23, 27, 30] were analyzed, respectively. Genotyping data obtained came from PCR-RFLP and TaqMan detection methods. Genotype and allele frequencies, sample size and other information were revealed in Table 1. Of the 11 studies, 4 presented a significant deviation from HWE (2 studies on rs4759314 [31, 32], 3 on rs920778 [23, 27, 33] and 2 on rs874945 [31, 32]). NOS scale in each study was assessed and the results showed that all the studies had high-quality (Table 2). Of the 11 studies, 2 populations including Chinese and Turks and 7 types of cancer involving ESCC, colorectal cancer, gastric cancer, breast cancer, osteosarcoma, EOC and cervical cancer were used for group analysis (Table 1).

**Table 1:** Characteristics of studies included in the meta-analysis

Author	Year	Country	Ethnicity	Cancer Type	Source of Control	Genetyping Method	Case/Control	Case			Control			Risk to cancer	P for HWE	Quality
rs4759314 A>G								AA	AG	GG	AA	AG	GG			
Zhang	2014	China	Asian	ESCC	HB	PCR-PFLP	1000/1000	917	81	2	910	89	1	none	0.436	8
Xue	2014	China	Asian	Colorectal cancer	HB	TaqMan	1733/1855	1528	200	5	1608	236	11	none	0.467	7
Guo	2015	China	Asian	GCA	HB	PCR-PFLP	515/654	461	53	1	589	64	1	none	0.587	7
Pan	2015	China	Asian	Gastric cancer	HB	PCR-PFLP	500/1000	451	48	1	914	83	3	none	0.448	7
Du	2015	China	Asian	Gastric cancer	HB	TaqMan	1275/1644	1083	186	6	1464	172	8	increased	0.230	8
Yan	2015	China	Asian	Breast cancer	PB	PCR-PFLP	502/504	451	50	1	448	54	2	none	0.785	7
Zhou	2016	China	Asian	Osteosarcoma	HB	TaqMan	500/500	423	62	15	425	64	11	none	<0.05	7
Wu	2016	China	Asian	EOC	HB	TaqMan	1000/1000	819	140	41	852	125	23	increased	<0.05	8
rs920778 C>T								CC	CT	TT	CC	CT	TT			
Zhang	2014	China	Asian	ESCC	HB	PCR-RFLP	2098/2150	1091	826	181	1323	749	78	increased	<0.05	8
Bayram	2015	Turkish	Caucasian	Breast cancer	HB	TaqMan	123/122	31	52	40	15	66	41	increased	0.140	6
Pan	2015	China	Asian	Gastric cancer	HB	PCR-RFLP	800/1600	420	321	59	980	575	45	increased	<0.05	7
Bayram	2015	Turkish	Caucasian	Gastric cancer	HB	TaqMan	104/209	20	52	32	38	105	66	none	0.738	6
Yan	2015	China	Asian	Breast cancer	PB	PCR-RFLP	502/504	12	151	339	18	190	296	increased	0.060	7
Qiu	2016	China	Asian	Cervical cancer	HB	TaqMan	215/430	90	78	47	226	150	54	increased	<0.05	7
rs7958904 G>C								GG	GC	CC	GG	GC	CC			
Xue	2014	China	Asian	Colorectal cancer	HB	TaqMan	1731/1852	1019	605	107	992	704	156	decreased	0.052	7
Du	2015	China	Asian	Gastric cancer	HB	TaqMan	739/1057	412	276	51	568	404	85	none	0.271	8
Zhou	2016	China	Asian	Osteosarcoma	HB	TaqMan	900/900	524	320	56	466	346	88	decreased	<0.05	7
Wu	2016	China	Asian	EOC	HB	TaqMan	1000/1000	594	355	51	533	380	87	decreased	0.105	8
rs874945 G>A								GG	GA	AA	GG	GA	AA			
Xue	2014	China	Asian	Colorectal cancer	HB	TaqMan	1147/1202	751	356	40	817	346	39	none	0.749	7
Du	2015	China	Asian	Gastric cancer	HB	TaqMan	751/1057	495	225	31	714	307	36	none	0.672	8
Zhou	2016	China	Asian	Osteosarcoma	HB	TaqMan	900/900	577	256	67	608	243	49	increased	<0.05	7
Wu	2016	China	Asian	EOC	HB	TaqMan	1000/1000	665	283	52	677	279	44	none	<0.05	8
rs1899663 G>T								GG	GA	AA	GG	GA	AA			
Zhang	2014	China	Asian	ESCC	HB	PCR-RFLP	1000/1000	725	256	19	724	250	26	none	0.430	7
Pan	2015	China	Asian	Gastric cancer	HB	PCR-RFLP	500/1000	376	118	6	732	255	13	none	0.078	7
Yan	2015	China	Asian	Breast cancer	PB	PCR-RFLP	502/504	339	149	14	326	158	20	none	0.876	7

ESCC: Esophageal squamous cell carcinoma; GCA: Gastric cardia adenocarcinoma; EOC: Epithelial ovarian cancer; HB: Hospital-based; PB: Population-based; HWE: Hardy–Weinberg equilibrium.

**Table 2:** Newcastle-ottawa quality assessment scale for each included study

Studies	Selection				Comparability		Exposure			Total quality score
	Case definition adequate	Representativeness of the cases	Selection of controls	Definition of controls	Adjustment for age	Adjustment for lifestyle/traditional risk factors	Ascertainment of exposure	Uniform method of ascertainment	Non-response rate	
Zhang 2014	1	1	1	1	1	1	0	1	1	8
Xue 2014	1	1	1	1	1	1	0	1	0	7
Guo 2015	1	1	1	1	1	1	0	1	0	7
Bayram 2015	1	1	0	1	1	1	0	1	0	6
Pan 2015	1	1	1	1	1	1	0	1	0	7
Bayram 2015	1	1	0	1	1	1	0	1	0	6
Du 2015	1	1	1	1	1	1	0	1	1	8
Yan 2015	1	1	1	1	1	1	0	1	0	7
Zhou 2016	1	1	1	1	1	1	0	1	0	7
Wu 2016	1	1	1	1	1	1	0	1	1	8
Qiu 2016	1	1	1	1	1	1	0	1	0	7

### Association between HOTAIR rs4759314 and cancer susceptibility

We analyzed the association between rs4759314 (A>G) and cancer susceptibility in 8 studies with 7025 cases and 8157 controls. As a result, we did not find a significant association between rs4759314 (A>G) and cancer susceptibility in any genetic model in general population. Moreover, we did not find a significant association between them in subgroup analysis of cancer type either (Table 3).

**Table 3:** Results of meta-analysis for rs4759314 and rs920778 polymorphisms and the risk of cancer

Locus	N	OR(95%CI)	P_h_	I^2^(%)	OR(95%CI)	P_h_	I^2^(%)	OR(95%CI)	P_h_	I^2^(%)	OR(95%CI)	P_h_	I^2^(%)	OR(95%CI)	P_h_	I^2^(%)
rs4759314 A>G		G VS A			GG VS AA			AG VS AA			AG/GG VS AA			GG VS AA/AG		
Overall	8	1.084(0.933-1.259)	0.018	58.5	1.298(0.916-1.840)	0.483	0.0	1.067(0.922-1.235)	0.065	47.4	1.080(0.929-1.225)	0.036	53.4	1.283(0.906-1.817)	0.504	0.0
Cancer Types																
Digestive cancer	5	1.060(0.861-1.305)	0.018	66.5	0.771(0.403-1.473)	0.778	0.0	1.080(0.866-1.348)	0.017	66.9	1.072(0.860-1.337)	0.015	67.6	0.761(0.398-1.456)	0.789	0.0
Other cancer	3	1.135(0.904-1.427)	0.160	45.4	1.625(1.064-2.482)	0.506	0.0	1.058(0.876-1.277)	0.555	0.0	1.115(0.915-1.359)	0.300	16.9	1.605(1.051-2.450)	0.531	0.0
rs920778 C>T		T VS C			TT VS CC			CT VS CC			CT/TT VS CC			TT VS CC/CT		
Overall	6	1.293(1.105-1.512)	0.002	73.0	1.675(1.034-2.714)	0.000	82.2	1.167(0.946-1.440)	0.029	59.8	1.278(1.025-1.593)	0.010	67.1	1.671(1.195-2.337)	0.000	79.2
Ethnicity																
Asian	4	1.464(1.362-1.574)	0.873	0.0	2.647(2.173-3.226)	0.452	0.0	1.322(1.197-1.459)	0.988	0.0	1.468(1.336-1.613)	0.980	0.0	2.081(1.523-2.845)	0.013	72.3
Caucasian	2	0.858(0.671-1.096)	0.321	0.0	0.672(0.349-1.293)	0.199	39.4	0.608(0.251-1.474)	0.064	70.8	0.633(0.287-1.397)	0.080	67.4	0.958(0.663-1.383)	0.976	0.0
Cancer Types																
Digestive cancer	3	1.367(1.165-1.605)	0.054	65.8	2.174(1.261-3.748)	0.008	79.4	1.314(1.186-1.455)	0.564	0.0	1.440(1.294-1.601)	0.339	7.6	1.941(1.120-3.364)	0.002	83.7
Other cancer	3	1.199(0.831-1.731)	0.004	82.4	1.248(0.505-3.084)	0.003	82.9	0.868(0.411-1.834)	0.010	78.2	1.012(0.451-2.271)	0.003	83.2	1.440(1.035-2.002)	0.123	52.3

N: the number of studies; P_h_: P- value for heterogeneity test.

### Association between HOTAIR rs920778 and cancer susceptibility

**Figure 2:**
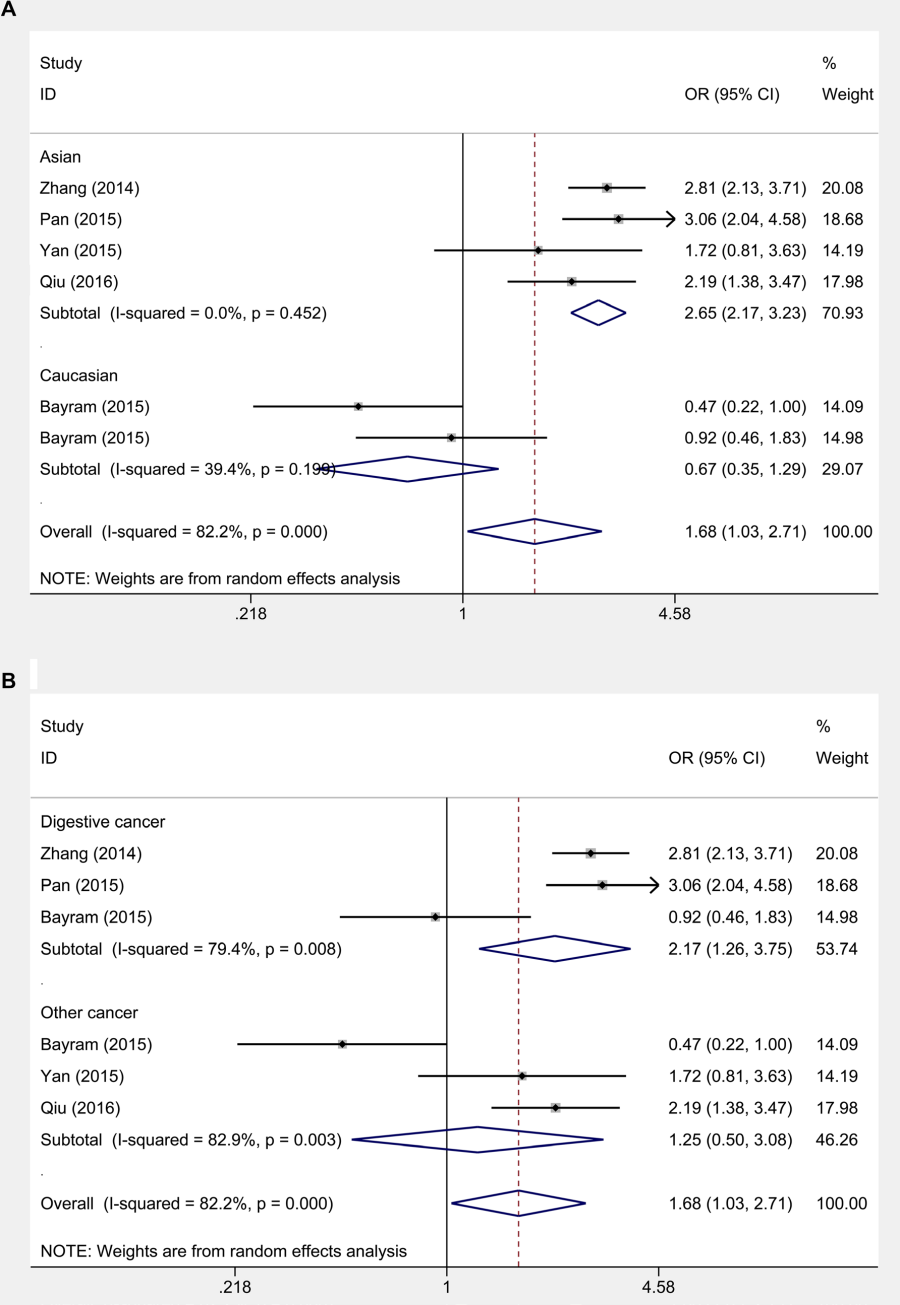
Subgroup analysis on relationship between HOTAIR rs920778 (C>T ) and cancer risk (**A**) Ethnicity analysis. (**B**) Cancer type analysis.

Association between rs920778 (C>T ) and cancer risk was analyzed in 6 studies with 3842 cases and 5015 controls. Overall, we observed a significantly increased risk of cancer susceptibility in homozygote comparison, dominant model and recessive model (TT versus CC: OR = 1.675, 95% CI 1.034–2.714, P_h_ < 0.01; CT/TT versus CC: OR = 1.278, 95% CI 1.025–1.593, P_h_ = 0.10; TT versus CC/CT: OR = 1.671, 95% CI 1.195–2.337, P_h_ < 0.01), but not in allele contrast model and heterozygote comparison (Table 3). In subgroup analysis, rs920778 (C>T ) showed a significant increased risk of cancer in all the genetic models in Asian population (T versus C: OR = 1.464, 95% CI 1.362–1.574, P_h_ = 0.873; TT versus CC: OR = 2.647, 95% CI 2.173–3.226, P_h_ = 0.452; CT versus CC: OR = 1.322, 95% CI 1.197–1.459, P_h_ = 0.988; CT/TT versus CC: OR = 1.468, 95% CI 1.336–1.613, P_h_ = 0.980; TT versus CC/CT: OR = 2.081, 95% CI 1.523–2.845, P_h_ = 0.013) (Figure 2A). In cancer type analysis, rs920778 (C>T) also revealed a significantly increased risk of digestive cancer in all the genetic models (T versus C: OR = 1.367, 95% CI 1.165–1.605, P_h_ = 0.054; TT versus CC: OR = 2.174, 95% CI 1.261–3.748, P_h_ = 0.008; CT versus CC: OR = 1.314, 95% CI 1.186–1.455, P_h_ = 0.564; CT/TT versus CC: OR = 1.440, 95% CI 1.294–1.601, P_h_ = 0.339; TT versus CC/CT: OR = 1.941, 95% CI 1.120–3.364, P_h_ = 0.002) (Figure 2B).

### Association between HOTAIR rs7958904, rs874945 or rs1899663 and cancer susceptibility

**Figure 3:**
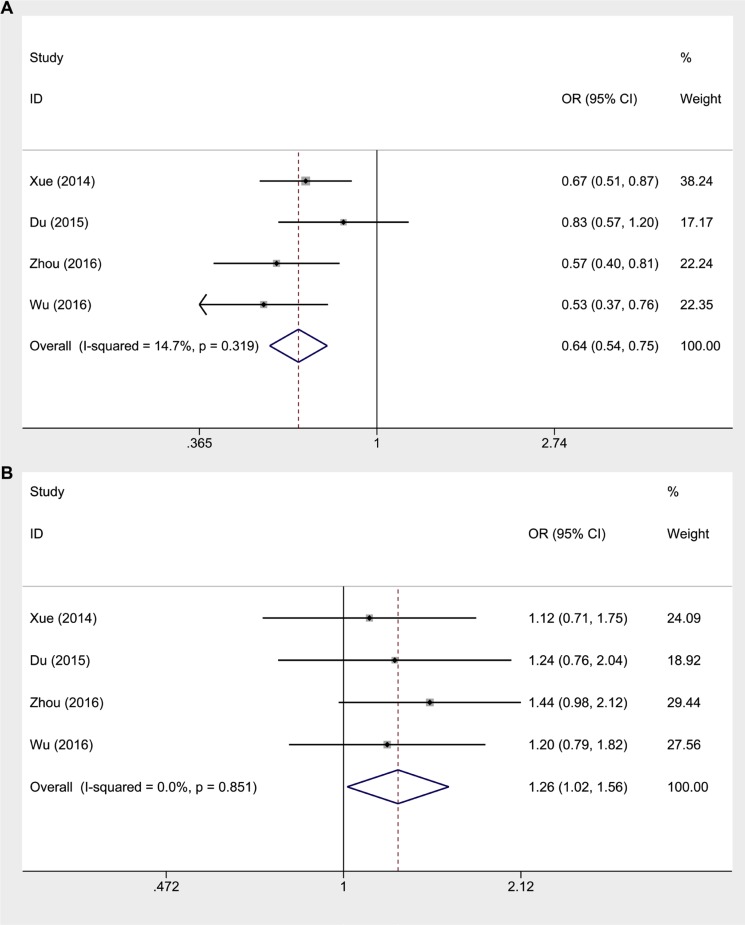
Forest plot of the association between rs7958904 or rs874945 and cancer risk in a homozygote comparison model (**A**) rs7958904 analysis result. (**B**) rs874945 analysis result.

Analysis results between HOTAIR rs7958904, rs874945 or rs1899663 and cancer risk were shown in Table 4. In general, there existed a significant association of rs7958904 polymorphism with decreased cancer risk in all genetic models (C versus G: OR = 0.818, 95% CI 0.766–0.875, P_h_ = 0.313; CC versus GG: OR = 0.641, 95% CI 0.544–0.755, P_h_ = 0.319; GC versus GG: OR = 0.853, 95% CI 0.782–0.931, P_h_ = 0.754; GC/CC versus GG: OR = 0.814, 95% CI 0.749–0.884, P_h_ = 0.339; CC versus GG/GC: OR = 0.682, 95% CI 0.582–0.801, P_h_ = 0.393) (Figure 3A). When analyzing rs874945, we observed a significantly increased cancer risk in allele contrast model, homozygote comparison and dominant model (A versus G: OR = 1.106, 95% CI 1.021–1.197, P_h_ = 0.816; AA versus GG: OR = 1.259, 95% CI 1.015–1.562, P_h_ = 0.851; GA/AA versus GG: OR = 1.104, 95% CI 1.005–1.212, P_h_ = 0.896), but not in heterozygote comparison and recessive model (Figure 3B). Similar to rs4759314, rs1899663 polymorphism was not in significant association with cancer risk in all genetic models (Table 4).

**Table 4:** Results of meta-analysis for rs7958904, rs874945 and rs1899663 polymorphisms and the risk of cancer

Locus	N	OR(95%CI)	P_h_	I^2^ (%)	OR(95%CI)	P_h_	I^2^(%)	OR(95%CI)	P_h_	I^2^(%)	OR(95%CI)	P_h_	I^2^(%)	OR(95%CI)	P_h_	I^2^(%)
rs7958904 G>C		C VS G			CC VS GG			GC VS GG			GC/CC VS GG			CC VS GG/GC		
Overall	4	0.818(0.766-0.875)	0.313	15.8	0.641(0.544-0.755)	0.319	14.7	0.853(0.782-0.931)	0.754	0.0	0.814(0.749-0.884)	0.523	0.0	0.682(0.582-0.801)	0.393	0.0
rs874945 G>A		A VS G			AA VS GG			GA VS GG			GA/AA VS GG			AA VS GG/GA		
Overall	4	1.106(1.021-1.197)	0.816	0.0	1.259(1.015-1.562)	0.851	0.0	1.081(0.980-1.193)	0.927	0.0	1.104(1.005-1.212)	0.896	0.0	1.231(0.944-1.523)	0.853	0.0
rs1899663 G>T		T VS G			TT VS GG			GT VS GG			GT/TT VS GG			TT VS GG/GT		
Overall	3	0.928(0.825-1.043)	0.774	0.0	0.737(0.487-1.114)	0.894	0.0	0.956(0.834-1.095)	0.675	0.0	0.937(0.821-1.069)	0.714	0.0	0.746(0.494-1.125)	0.891	0.0

N: the number of studies; P_h_: P- value for heterogeneity test.

### Sensitivity analysis result

Sensitivity analysis was used to evaluate individual study's influence on the pooled results by deleting one single study each time from pooled analysis. As a result, rs920778 in Bayram's study [28] had a significant effect on the pooled OR. When this study was excluded in the genotype comparison of TT versus CC, the heterogeneity test was significantly reduced (data not shown).

### Publication bias analysis result

**Figure 4:**
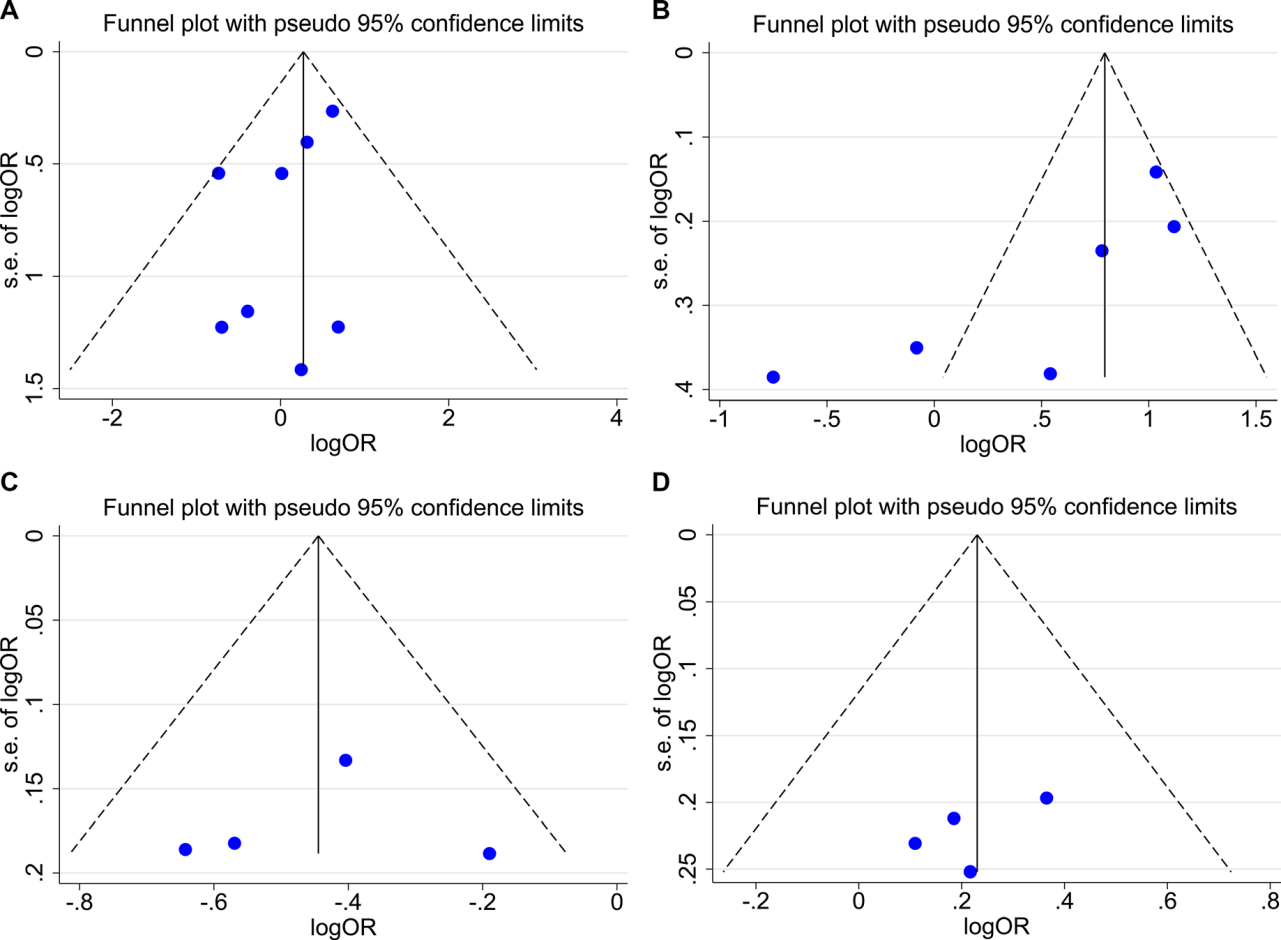
Funnel plot for publication bias test (**A**) rs4759314 analysis result. (**B**) rs920778 analysis result. (**C**) rs795904 analysis result. (**D**) rs874945 analysis result.

A funnel plot was generated to assess potential publication bias. As shown in Figure 4, there was no any evidence of publication bias in rs4759314, rs7958904 or rs874945 or rs1899663 analysis. Result of publication bias was not shown. There was only slight statistical evidence of publication bias in rs920778 analysis (*P*_b_ = 0.024, *P*_e_ = 0.040).

## DISCUSSION

In the study, five potential HOTAIR SNPs including rs4759314, rs920778, rs7958904, rs874945 and rs1899663 susceptible to cancer were identified and used for meta-analysis. SNP rs920778, rs4759314 and rs1899663 are located in the introns, rs7958904 in the coding region and rs874945 is close to 3′ region of the HOTAIR gene, respectively [24].

Overall, our results provide evidences that rs920778, rs7958904 and rs874945 but not rs4759314 and rs1899663 loci are related to cancer risk, among of which rs920778 and rs874945 increase and rs7958904 decreases cancer risk, respectively. In the study, we also found that rs920778 is susceptible to cancer in Asian population but not in Turks, which is in accord with Tian's study [20]. We speculate that there are two reasons to explain the difference. First, different genetic backgrounds may contribute to divergence because the distribution of HOTAIR allele frequency varies among Asians and Caucasians. Second, different populations have various lifestyles and are influenced by different environmental factors.

Previously, four papers have discussed the association of HOTAIR polymorphisms with cancer risk by meta-analysis [20–22, 34]. In comparison with these studies, our meta-analysis has the following characteristics (Table 5). In the study, we have selected 11 related articles more than that in any study mentioned above. The enrolled SNPs and cases including patients and controls in our study are also much more than those in any study, implying that our assessment of the relationship between the HOTAIR gene SNPs and cancer risk is relatively more precise. As for rs920778 polymorphism, all five meta-analysis studies including ours have same results which indicate that the polymorphism can increase cancer risk, especially in Asian population and digestive cancer. However, Bayram et al. did not find association of rs920778 to gastric cancer in a Turkish population by a case-control study [28]. For SNP rs4759314, all five meta-analysis studies showed that the SNP is not a risk factor of cancer. For rs1899663, our study together with other three studies also obtained the negative association of the polymorphic site to cancer risk. Same to Zhang's group study [21], our group study showed that rs7958904 site decreases cancer risk. In addition, we first analyzed the association of rs874945 to cancer risk. As a result, the polymorphic site decreases the risk of cancer.

**Table 5:** The characteristics and comparison of different meta-analysis

Author (Other meta analysis)	Enrolled papers	Case/Control	genetic model	enrolled SNPs	overall risk to cancer	subgroup (ethnicity)	subgroup (cancer type)
Tian	8	7151/8740	three models: heterozygote comparison, homozygote comparison, dominant model	rs920778	increased risk	Asian	digestive cancer
			One model: dominant model	rs4759314	none	none	none
			One model: dominant model	rs1899663	none	none	none
Zhang	6		five models: allele contrast model, dominant model, recessive model, heterozygote comparison, homozygote comparison	rs920778	increased risk	Asian	none
			five models: allele contrast model, dominant model, recessive model, heterozygote comparison, homozygote comparison	rs4759314	none	none	none
			five models: allele contrast model, dominant model, recessive model, heterozygote comparison, homozygote comparison	rs1899663	none	none	none
Qi	9	7772/9075	five models: allele contrast model, dominant model, recessive model, heterozygote comparison, homozygote comparison	rs920778	increased risk	Asian	gastric cancer
			five models: allele contrast model, dominant model, recessive model, heterozygote comparison, homozygote comparison	rs4759314	none	none	none
Zhang	8	7151/8740	four models: allele contrast model, dominant model, recessive model, homozygote comparison	rs920778	increased risk	Asian	gastric cancer
			four models: allele contrast model, dominant model, recessive model, homozygote comparison	rs4759314	none	none	none
			four models: allele contrast model, dominant model, recessive model, homozygote comparison	rs1899663	none	none	none
			four models: allele contrast model, dominant model, recessive model, homozygote comparison	rs7958904	decreased risk	none	none
Present study	11	10867/13172	five models: allele contrast model, dominant model, recessive model, heterozygote comparison, homozygote comparison	rs920778	increased risk	Asian	digestive cancer
			five models: allele contrast model, dominant model, recessive model, heterozygote comparison, homozygote comparison	rs4759314	none	none	none
			five models: allele contrast model, dominant model, recessive model, heterozygote comparison, homozygote comparison	rs1899663	none	none	none
			five models: allele contrast model, dominant model, recessive model, heterozygote comparison, homozygote comparison	rs7958904	decreased risk	none	none
			five models: allele contrast model, dominant model, recessive model, heterozygote comparison, homozygote comparison	rs874945	increased risk	none	none

Zhang et al. identified a intronic enhancer between +1719bp and +2353bp from the transcriptional start site of HOTAIR gene and the SNP rs920778 is just located in the region. Moreover, they found that the SNP can increase HOTAIR RNA expression by altering the enhancer activity in esophageal squamous cell carcinoma [23]. As a SNP in the coding sequence, rs7958904 does not alter HOTAIR transcript, but it is predicted to change the secondary structure of HOTAIR, indicating that the SNP deregulates HOTAIR expression by affecting the gene structure, leading to carcinogenesis [24]. SNP rs874945 is near 3′ region of the HOTAIR gene. It is well known that gene 3′ region is a regulatory target of coding gene products [35]and non-coding genes such as microRNA [36]. It has been reported that SNP rs1126579 in the CXCR2 gene 3′ region disrupts the binding site for miR-516a-3p, lead to a moderate increase in CXCR2 mRNA and protein expression, and increased MAPK signaling [37]. Even though SNP rs874945 functional study is not reported, we speculate that it functions by interfering the bind with other coding gene products and non-coding genes.

However, there are several limitations to the present study. Few studies were included in this meta-analysis, and this small sample size limits the power to detect the associations. Because the power of funnel plots, Egger's and Begg's test of publication bias may also greatly constrain our analysis, our conclusions should be interpreted cautiously. For examples, there was a slight publication bias in the studies on rs920778. We think that only English language studies selected in the study partially contributes the bias. In addition, subgroup analyses of the rs7958904, rs874945 and rs1899663 loci were not performed because the sample size in each study was relatively not large enough.

Although HOTAIR polymorphisms are associated with assessment of cancer risk, abnormal expression of HOTAIR itself is also useful in predicting cancer prognosis. For examples, high HOTAIR level is correlated to poor prognosis in digestive system tumors [38], estrogen-dependent malignant tumors [39], breast cancer, non-small cell lung cancer, laryngeal carcinoma [40], diffuse large B cell lymphoma [41], bladder transitional cell carcinoma [42] and acute leukemia [43]. Thus, HOTAIR is a key LncRNA in genesis and development of cancer.

In conclusion, HOTAIR SNP rs920778, rs7958904 and rs874945 are cancer risk factors, among of which rs920778 is also useful in evaluating cancer risk of Asian population and digestive cancer. Moreover, rs874945 as a cancer risk SNP is first reported in the meta-analysis.

## MATERIALS AND METHODS

### Identification of eligible studies and data extraction

We performed a publication search using PubMed, Embase and Web of Science updated on June 29,2016. The following search terms were used: “HOTAIR OR HOX transcript antisense intergenic RNA”,”polymorphism OR variation OR variant OR mutation”, “cancer OR carcinoma OR tumor OR tumour OR neoplasm”, respectively. Searching was done without restriction on language or publication years. Inclusion criteria for studies were as followings: (1) articles on HOTAIR polymorphisms and cancer risk; (2) case-control studies; (3) studies that had detailed genotype frequency of cases and controls or could be calculated from the paper text. Exclusion criteria included: (1) abstract, comment, review; (2) duplication of the previous publications; (3) lack of usable genotype frequency data.

### Data extraction

Based on the inclusion criteria, two reviewers (ZX Zhang and WN Fu) independently extracted information from all eligible publications. The following information were included in each study: name of first author, year of publication, country, ethnicity, cancer types, source of control, genotyping method, numbers of cases and controls, allele as well as genotype frequencies for cases and controls, *P* value for Hardy–Weinberg equilibrium (HWE), article quality. Different ethnicity descents were categorized as Asians or Caucasians. Study design was stratified into population-based and hospital-based studies. Any disagreement was resolved through discussion until the two reviewers reached a consensus.

### Quality score assessment

Two reviewers (ZX Zhang and X Tong) independently assessed the quality of the included studies according to the Newcastle Ottawa Scale (NOS) (http://www.ohri.ca/programs/clinical_epidemiology/oxford.asp). The scale consists of three components related to sample selection, comparability and ascertainment of exposure.

### Statistics analysis

HWE was evaluated using Chi-square test in control groups of each study. Strength of association between HOTAIR SNPs and cancer risk was assessed by odds ratios (ORs) with 95% confidence intervals (CIs). Statistical significance of the pooled OR was determined by Z test. Pooled ORs were used to calculate allele frequency comparison ( rs4759314: G versus A, rs920778: T versus C, rs7958904: C versus G, rs874945: A versus G,rs1899663: T versus G). Five different ORs were calculated according to allele contrast model, dominant model, recessive model, heterozygote comparison and homozygote comparison. Subgroup analyses were performed by cancer types and ethnicities. Heterogeneity degree between different studies was determined by Q-statistic [44, 45]. If there was no significance in heterogeneity degree, the fixed effect model (Mantel-Haenszel method) would be used [46]. Otherwise, the random effect model (DerSimonian and Laird method) would be used [47]. Heterogeneity effect was then quantified by I^2^ test. I^2^ statistics represented the proportion of variation across studies due to between-study heterogeneity, and the values of 25%, 50% and 75% were regarded as cut-off points for low, moderate and high degrees of heterogeneity, respectively [44]. Sensitivity analysis was performed to evaluate the stability of the results. Publication bias was evaluated by funnel plot and quantified by Begg's test and Egger's test to assess funnel plot asymmetry [48, 49]. Meta-analyses were performed with the software Stata (Version 12, College Station, Texas, USA). All statistical tests were two-sided and *p* < 0.05 was considered statistically significant.

## References

[1] Esteller M (2011). Non-coding RNAs in human disease. Nat Rev Genet.

[2] Lee JT (2012). Epigenetic regulation by long noncoding RNAs. Science.

[3] Kung JT, Colognori D, Lee JT (2013). Long noncoding RNAs: past, present, and future. Genetics.

[4] Tran NT, Su H, Khodadadi-Jamayran A, Lin S, Zhang L, Zhou D, Pawlik KM, Townes TM, Chen Y, Mulloy JC, Zhao X (2016). The AS-RBM15 lncRNA enhances RBM15 protein translation during megakaryocyte differentiation. Embo Rep.

[5] Yang F, Bi J, Xue X, Zheng L, Zhi K, Hua J, Fang G (2012). Up-regulated long non-coding RNA H19 contributes to proliferation of gastric cancer cells. FEBS J.

[6] Pickard MR, Mourtada-Maarabouni M, Williams GT (2013). Long non-coding RNA GAS5 regulates apoptosis in prostate cancer cell lines. Biochim Biophys Acta.

[7] Ellis BC, Graham LD, Molloy PL (2014). CRNDE, a long non-coding RNA responsive to insulin/IGF signaling, regulates genes involved in central metabolism. Biochim Biophys Acta.

[8] Wang K, Liu CY, Zhou LY, Wang JX, Wang M, Zhao B, Zhao WK, Xu SJ, Fan LH, Zhang XJ, Feng C, Wang CQ, Zhao YF (2015). APF lncRNA regulates autophagy and myocardial infarction by targeting miR-188–3p. Nat Commun.

[9] Guo H, Ahmed M, Zhang F, Yao CQ, Li S, Liang Y, Hua J, Soares F, Sun Y, Langstein J, Li Y, Poon C, Bailey SD (2016). Modulation of long noncoding RNAs by risk SNPs underlying genetic predispositions to prostate cancer. Nat Genet.

[10] Yuan H, Liu H, Liu Z, Owzar K, Han Y, Su L, Wei Y, Hung RJ, McLaughlin J, Brhane Y, Brennan P, Bickeboeller H, Rosenberger A (2016). A Novel Genetic Variant in Long Non-coding RNA Gene NEXN-AS1 is Associated with Risk of Lung Cancer. Sci Rep.

[11] Yao L, Tak YG, Berman BP, Farnham PJ (2014). Functional annotation of colon cancer risk SNPs. Nat Commun.

[12] Cowper-Sal LR, Zhang X, Wright JB, Bailey SD, Cole MD, Eeckhoute J, Moore JH, Lupien M (2012). Breast cancer risk-associated SNPs modulate the affinity of chromatin for FOXA1 and alter gene expression. Nat Genet.

[13] Myles S, Davison D, Barrett J, Stoneking M, Timpson N (2008). Worldwide population differentiation at disease-associated SNPs. Bmc Med Genomics.

[14] Rinn JL, Kertesz M, Wang JK, Squazzo SL, Xu X, Brugmann SA, Goodnough LH, Helms JA, Farnham PJ, Segal E, Chang HY (2007). Functional demarcation of active and silent chromatin domains in human HOX loci by noncoding RNAs. Cell.

[15] Chiyomaru T, Fukuhara S, Saini S, Majid S, Deng G, Shahryari V, Chang I, Tanaka Y, Enokida H, Nakagawa M, Dahiya R, Yamamura S (2014). Long non-coding RNA HOTAIR is targeted and regulated by miR-141 in human cancer cells. J Biol Chem.

[16] Gupta RA, Shah N, Wang KC, Kim J, Horlings HM, Wong DJ, Tsai MC, Hung T, Argani P, Rinn JL, Wang Y, Brzoska P, Kong B (2010). Long non-coding RNA HOTAIR reprograms chromatin state to promote cancer metastasis. Nature.

[17] Kreile M, Piekuse L, Rots D, Dobele Z, Kovalova Z, Lace B (2016). Analysis of possible genetic risk factors contributing to development of childhood acute lymphoblastic leukaemia in the Latvian population. Arch Med Sci.

[18] Grotenhuis AJ, Dudek AM, Verhaegh GW, Aben KK, Witjes JA, Kiemeney LA, Vermeulen SH (2016). Independent Replication of Published Germline Polymorphisms Associated with Urinary Bladder Cancer Prognosis and Treatment Response. Bl Cancer.

[19] Murali A, Varghese BT, Kumar RR, Kannan S (2016). Combination of genetic variants in cyclin D1 and retinoblastoma genes predict clinical outcome in oral cancer patients. Tumour Biol.

[20] Tian T, Li C, Xiao J, Shen Y, Lu Y, Jiang L, Zhuang X, Chu M (2016). Quantitative Assessment of the Polymorphisms in the HOTAIR lncRNA and Cancer Risk: A Meta-Analysis of 8 Case-Control Studies. Plos One.

[21] Zhang J, Liu X, You LH, Zhou RZ (2016). Significant association between long non-coding RNA HOTAIR polymorphisms and cancer susceptibility: a meta-analysis. Onco Targets Ther.

[22] Qi Q, Wang J, Huang B, Chen A, Li G, Li X, Wang J (2016). Association of HOTAIR polymorphisms rs4759314 and rs920778 with cancer susceptibility on the basis of ethnicity and cancer type. Oncotarget.

[23] Zhang X, Zhou L, Fu G, Sun F, Shi J, Wei J, Lu C, Zhou C, Yuan Q, Yang M (2014). The identification of an ESCC susceptibility SNP rs920778 that regulates the expression of lncRNA HOTAIR via a novel intronic enhancer. Carcinogenesis.

[24] Xue Y, Gu D, Ma G, Zhu L, Hua Q, Chu H, Tong N, Chen J, Zhang Z, Wang M (2015). Genetic variants in lncRNA HOTAIR are associated with risk of colorectal cancer. Mutagenesis.

[25] Guo W, Dong Z, Bai Y, Guo Y, Shen S, Kuang G, Xu J (2015). Associations between polymorphisms of HOTAIR and risk of gastric cardia adenocarcinoma in a population of north China. Tumour Biol.

[26] Bayram S, Sumbul AT, Batmaci CY, Genc A (2015). Effect of HOTAIR rs920778 polymorphism on breast cancer susceptibility and clinicopathologic features in a Turkish population. Tumour Biol.

[27] Pan W, Liu L, Wei J, Ge Y, Zhang J, Chen H, Zhou L, Yuan Q, Zhou C, Yang M (2016). A functional lncRNA HOTAIR genetic variant contributes to gastric cancer susceptibility. Mol Carcinog.

[28] Bayram S, Ulger Y, Sumbul AT, Kaya BY, Rencuzogullari A, Genc A, Sevgiler Y, Bozkurt O, Rencuzogullari E (2015). A functional HOTAIR rs920778 polymorphism does not contributes to gastric cancer in a Turkish population: a case-control study. Fam Cancer.

[29] Du M, Wang W, Jin H, Wang Q, Ge Y, Lu J, Ma G, Chu H, Tong N, Zhu H, Wang M, Qiang F, Zhang Z (2015). The association analysis of lncRNA HOTAIR genetic variants and gastric cancer risk in a Chinese population. Oncotarget.

[30] Yan R, Cao J, Song C, Chen Y, Wu Z, Wang K, Dai L (2015). Polymorphisms in lncRNA HOTAIR and susceptibility to breast cancer in a Chinese population. Cancer Epidemiol.

[31] Zhou Q, Chen F, Fei Z, Zhao J, Liang Y, Pan W, Liu X, Zheng D (2016). Genetic variants of lncRNA HOTAIR contribute to the risk of osteosarcoma. Oncotarget.

[32] Wu H, Shang X, Shi Y, Yang Z, Zhao J, Yang M, Li Y, Xu S (2016). Genetic variants of lncRNA HOTAIR and risk of epithelial ovarian cancer among Chinese women. Oncotarget.

[33] Qiu H, Liu Q, Li J, Wang X, Wang Y, Yuan Z, Li J, Pei DS (2016). Analysis of the association of HOTAIR single nucleotide polymorphism (rs920778) and risk of cervical cancer. APMIS.

[34] Zhang C, Shen M, Xu X, Hu Y, Zhang Z, Duan H, Niu Y, Yuan H (2016). Quantitative assessment of the association between functional long non-coding RNA HOTAIR genetic variants and cancer susceptibility. Int J Clin Exp Med.

[35] Palanichamy JK, Tran TM, Howard JM, Contreras JR, Fernando TR, Sterne-Weiler T, Katzman S, Toloue M, Yan W, Basso G, Pigazzi M, Sanford JR, Rao DS (2016). RNA-binding protein IGF2BP3 targeting of oncogenic transcripts promotes hematopoietic progenitor proliferation. J Clin Invest.

[36] Shen CT, Qiu ZL, Song HJ, Wei WJ, Luo QY (2016). miRNA-106a directly targeting RARB associates with the expression of Na(+)/I(−) symporter in thyroid cancer by regulating MAPK signaling pathway. J Exp Clin Cancer Res.

[37] Ryan BM, Robles AI, McClary AC, Haznadar M, Bowman ED, Pine SR, Brown D, Khan M, Shiraishi K, Kohno T, Okayama H, Modali R, Yokota J (2015). Identification of a functional SNP in the 3′UTR of CXCR2 that is associated with reduced risk of lung cancer. Cancer Res.

[38] Ma G, Wang Q, Lv C, Qiang F, Hua Q, Chu H, Du M, Tong N, Jiang Y, Wang M, Zhang Z, Wang J, Gong W (2015). The prognostic significance of HOTAIR for predicting clinical outcome in patients with digestive system tumors. J Cancer Res Clin Oncol.

[39] Li J, Wen W, Zhao S, Wang J, Chen J, Wang Y, Zhang Q (2015). Prognostic role of HOTAIR in four estrogen-dependent malignant tumors: a meta-analysis. Onco Targets Ther.

[40] Zhang S, Chen S, Yang G, Gu F, Li M, Zhong B, Hu J, Hoffman A, Chen M (2014). Long noncoding RNA HOTAIR as an independent prognostic marker in cancer: a meta-analysis. Plos One.

[41] Yan Y, Han J, Li Z, Yang H, Sui Y, Wang M (2016). Elevated RNA expression of long noncoding HOTAIR promotes cell proliferation and predicts a poor prognosis in patients with diffuse large B cell lymphoma. Mol Med Rep.

[42] Shang C, Guo Y, Zhang H, Xue YX (2016). Long noncoding RNA HOTAIR is a prognostic biomarker and inhibits chemosensitivity to doxorubicin in bladder transitional cell carcinoma. Cancer Chemother Pharmacol.

[43] Zhang YY, Huang SH, Zhou HR, Chen CJ, Tian LH, Shen JZ (2016). Role of HOTAIR in the diagnosis and prognosis of acute leukemia. Oncol Rep.

[44] Higgins JP, Thompson SG, Deeks JJ, Altman DG (2003). Measuring inconsistency in meta-analyses. BMJ.

[45] Zintzaras E, Ioannidis JP (2005). Heterogeneity testing in meta-analysis of genome searches. Genet Epidemiol.

[46] Mantel N, Haenszel W (1959). Statistical aspects of the analysis of data from retrospective studies of disease. J Natl Cancer Inst.

[47] DerSimonian R, Laird N (1986). Meta-analysis in clinical trials. Control Clin Trials.

[48] Begg CB, Mazumdar M (1994). Operating characteristics of a rank correlation test for publication bias. Biometrics.

[49] Egger M, Davey SG, Schneider M, Minder C (1997). Bias in meta-analysis detected by a simple, graphical test. BMJ.

